# Survey of Infectious Etiologies of Bovine Abortion during Mid- to Late Gestation in Dairy Herds

**DOI:** 10.1371/journal.pone.0091549

**Published:** 2014-03-24

**Authors:** Mohamed Barkallah, Yaakoub Gharbi, Amal Ben Hassena, Ahlem Ben Slima, Zouhir Mallek, Michel Gautier, Gilbert Greub, Radhouane Gdoura, Imen Fendri

**Affiliations:** 1 Unité de recherche Toxicologie – Microbiologie Environnementale et Santé (UR11ES70), Faculté des Sciences de Sfax, Université de Sfax-Tunisia, Sfax, Tunisia; 2 Centre Vétérinaire de Recherche, Sfax, Tunisia; 3 Laboratoire de Microbiologie, Département agroalimentaire, Agrocampus Ouest, Rennes, France; 4 Institute of Microbiology, University Hospital Center and University of Lausanne, Lausanne, Switzerland; Cornell University, United States of America

## Abstract

Bovine abortion of unknown infectious etiology still remains a major economic problem. Thus, we investigated whether *Brucella* spp., *Listeria monocytogenes*, *Salmonella* spp., *Campylobacter* spp. and *Coxiella burnetii* are associated with abortion and/or stillbirth in Tunisian dairy cattle. Using a pan-*Chlamydiales* PCR, we also investigated the role of *Chlamydiaceae*, *Waddlia chondrophila, Parachlamydia acanthamoebae* and other members of the *Chlamydiales* order in this setting. Veterinary samples taken from mid to late-term abortions from twenty dairy herds were tested. From a total of 150 abortion cases collected, infectious agents were detected by PCR in 73 (48.66%) cases, 13 (8.66%) of which represented co-infections with two infectious agents. Detected pathogens include *Brucella spp* (31.3%), *Chlamydiaceae* (4.66%), *Waddlia chondrophila* (8%), *Parachlamydia acanthamoebae* (5.33%), *Listeria monocytogenes* (4.66%) and *Salmonella* spp. (3.33%). In contrast, *Campylobacter* spp. and *Coxiella burnetii* DNA were not detected among the investigated veterinary samples. This demonstrates that different bacterial agents may cause bovine abortion in Tunisia. This is the first report suggesting the role of *Parachlamydia acanthamoebae* in bovine abortion in Africa. Further studies with a larger number of samples are necessary to confirm whether this emerging pathogen is directly linked to abortion in cattle.

## Introduction

Abortion among dairy cows is one of the major causes of economic losses in the cattle industry. Abortions may be idiopathic or the result of metabolic or hormonal abnormalities, nutritional deficiencies, trauma, toxicities, or infectious agents. The latter represents the leading etiology of reproductive disorders [1], [2] A variety of infectious agents have been reported to cause bovine abortion throughout the world. The major bacterial agents that have been implicated in bovine abortion during mid- to late-gestation are *Brucella* spp., *Chlamydia* spp., *Salmonella* spp., *Campylobacter* spp., *Listeria monocytogenes* and *Coxiella burnetii* [3]–[5]. Bovine brucellosis was distributed worldwide, and its importance is related not only to the economic losses in animal production but also to a significant risk for human health. In cattle, brucellosis is caused by *Brucella abortus*, a facultative intracellular Gram negative coccobacillus. The abortion generally occurs from 6 to 9 months of gestation. It is frequently followed by fetal membrane retention and endometritis, which may be the cause of infertility in subsequent pregnancies [6], [7]. Even in the absence of abortion, excretion of the infectious organism occurs, for instance via placenta, foetal fluids, vaginal discharge, and milk. In addition to brucellosis, chlamydiosis and Q fever should be considered among the most common zoonotic diseases and are distributed all around the world. They are respectively caused by *Chlamydia* and *C. burnetii*, two strictly intracellular Gram negative bacteria. In cattle, *C. burnetii* infection is associated with abortions and stillbirths even if the infection is often asymptomatic. Inversely, chlamydial infections in cattle cause a variety of syndromes such as conjunctivitis, polyarthritis, encephalomyelitis, mastitis, infertility, abortion and other urogenital tract infections [8], [9]. *C. abortus*, *C. pecorum* and *W. chondrophila* are additional etiologies of chlamydial abortions in cattle [10]–[12]. Moreover, there is increasing evidences supporting the role of another *Chlamydia*-related bacteria, *P. acanthamoebae*, in abortion in both cattle [13]–[15] and humans [16]. However, no information is available regarding the presence of this bacterium in Africa.

Listeriosis, salmonellosis and campylobacteriosis are also serious zoonotic diseases. Bovine listeriosis is caused by *L. monocytogenes* and the organism is excreted in feces, urine and milk. Abortions are usually sporadic but may affect 10–20% of a herd. Abortions occur most commonly during the last trimester of pregnancy. The aborted fetus is often autolyzed. Bovine salmonellosis is caused predominantly by *S. enterica* serotypes Typhimurium and Dublin [17], [18]. Occasionally, *Salmonella* spp. cause abortion storms. The cows are usually sick and the fetuses and placentas are autolyzed and emphysematous. *Salmonella* can be isolated from the fetal tissues, vaginal fluids, feces and milk. Several *Campylobacter* species can be associated with abortion in cattle. Bovine genital campylobacteriosis is a venereal disease caused by *Campylobacter fetus* subsp. *venerealis* that can be found in the genital tract of cattle, in which it may cause genital tract infection and sporadic abortions [19].

Bacteriological isolation by culture on blood agar is usually used for the diagnosis of bovine brucellosis, but it is difficult, time consuming, hazardous, and sometimes inconclusive [20], [21]. Routine diagnosis of Q fever is often made by the use of serological tests [22], which have the disadvantage of indicating post-exposure rather than ongoing infection. Diagnosis of chlamydial infections in animals still represents a considerable challenge [23]. Isolation in cell culture remains difficult, time consuming, and depends on the presence of sufficient numbers of viable bacteria. The reliability of standard diagnostic procedures for *Campylobacter fetus*, which are based on phenotypic methods, is difficult because of specific nutritional and atmospheric requirements [24]. Furthermore, all these traditional culture and serology-based methods can be relatively insensitive depending on the quality and timing of sampling. Rapid detection of abortigenic agents at the early stage of an outbreak using molecular methods contributes to minimizing the spread of infection, the outbreak burden and increasing treatment efficiency. Therefore, real-time PCR have been increasingly used as a diagnostic tool for etiologic diagnosis of abortion in cattle [3], [15], [25], as a complement or replacement of time consuming traditional diagnostic methods such as bacterial culture [26].

Thus, the present study aimed (i) to investigate the role of some abortigenic agents in cattle from different geographical regions of Sfax in Tunisia (ii) to define the role of *P. acanthomoebae* as a new abortigenic agent in African cattle and (iii) to detect a possible co-infection of members of the *Chlamydiales* order with other abortigenic agents.

## Materials and Methods

### Animals and samples

Twenty dairy herds from different regions of Sfax (Tunisia) that experienced reproductive disorders (mainly abortions) from October 2010 to May 2012 were included in this study. Informations on individual animal such as age, sex and abortion history were recorded separately on sample data sheets. Herd sizes ranged from 20 to 1500 cows, knowing that all cows studied in this work are the descendents of pure Holsteins from the national production. All these dairy cows were vaccinated against foot and mouth disease (FMD) during national immunization campaigns. The majority of included cows were kept on limited pastures or tethered on a pasture. Seventy percent of the herds possessed small ruminants, and only twenty percent reported having bought new cattle. In dairy herds, nutrient requirements may not be the same depending on the animal's age and stage of production. Forages, which refer especially to hay or straw, are the most common type of feed used. Barley is an example of cereal grain that is extensively used in these herds. In all herds, samples from (i) cows with clinical signs (cases) and (ii) cows with normal pregnancies and normal parturitions (controls) were taken. A total of 214 animals were sampled: 150 cases and 64 controls. They were bled on the same day or up to 8 months after abortion. A total of 214 blood, 214 vaginal swabs and 214 milk samples were collected by local veterinarians and sent to the laboratory. Practically, blood samples of about 5 ml were aseptically collected using plain tubes from cows through jugular venipuncture. Serum samples were separated within 12 h of collection and transported to the laboratory using an ice box where they are stored at −20°C until tested. Vaginal swab samples were collected from each cow, using sterile swabs after vulva disinfection with chlorhexidine solution. All swabs were put into tubes containing 1 ml of 2-sucrose phosphate medium (2-SP). They were kept at 4°C during transportation in ice-pack containers, and then stored at −80°C until use. All milk samples were stored at −80°C until tested.

### DNA extraction and real-time PCR

The vaginal swab samples collected in 2-SP medium were thawed; 1 ml was transferred to a new microtube, and then centrifuged at 13,000 g for 20 min. The pellet was resuspended in 200 µl sterile water. The total volume was extracted by Quick-gDNA MiniPrep D3006 Kit (Zymo Research, Irvine, CA, USA) as recommended by the manufacturer. Extracted DNA was re-suspended in 50 µl of elution buffer and stored at −20°C until subsequent analysis.

For milk samples, DNA was extracted by using 1 ml aliquot which was centrifuged at 6,000× *g* for 10 min. The clear whey portion was suctioned out with a transfer pipette and discarded. The remaining milk solids and butterfat were resuspended in 200 µl sterile water and used for DNA extraction using Quick-gDNA MiniPrep D3006 Kit (Zymo Research, Irvine, CA, USA) as recommended by the manufacturer. Extracted DNA was re-suspended in 50 µl of elution buffer and stored at −20°C until subsequent analysis. All samples were tested in duplicate.

### *Brucella* spp. PCR

The real-time PCR assay was performed on a CFX96™ real-time PCR cycler (Biorad, Hercules, CA, USA). Real-time amplifications were carried out in a total reaction volume of 20 µl containing 10 µl iTaq Supermix with ROX (Bio-Rad, Reinach, Switzerland), 0.3 µM of each primer, 0.2 µM of probe and 5 µl of purified DNA to a final volume of 20 µl using nuclease-free water. Primers and probe sequences are provided in Table 1. The optimal PCR efficacy was obtained using a cycling profile that included an initial denaturation step at 95°C for 3 min, then 45 cycles of 15 s at 95°C and 1 min at 60°C. In all experiments, each PCR run included a negative extraction control (sterile water) and a negative PCR control containing 5 µl Diethylpyrocarbonate (DEPC) treated H_2_O instead of DNA extract, to detect possible contamination of DNA. Samples were run in duplicate.

**Table 1:** Sequences of primers and probes used in the study.

	Name	Sequence (5′ to 3′)	Product size (bp)	Reference
*Brucella* spp.	IS711-F	GCTTGAAGCTTGCGGACAGT	63	This study
	IS711-R	GGCCTACCGCTGCGAAT		
	IS711-P	HEX-AAGCCAACACCCGGCCATTATGGT-BHQ1		
*Chlamydiale* order	panCh16F2	CCGCCAACACTGGGACT	207 to 215	Lienard et al., 2011
	panCh16R2	GGAGTTAGCCGGTGCTTCTTTAC		
	panCh16S	FAM-CTACGGGAGGCTGCAGTCGAGAATC-BHQ1		
*Salmonella* spp.	invA-F	AGACGACTGGTACTGATTGATAAT	243	This study
	invA-R	ACAGTGCTCGTTTACGACCTGAAT		
*L. monocytogenes*	hly-F	CATGGCACCACCAGCATCT	63	This study
	Hly-R	ATCCGCGGTGTTTCTTTTCGA		
*C. burnetii*	IS1111-F	GCGTCATAATGCGCCAACATA	201	This study
	IS1111-R	CGCAGCCCACCTTAAGACTG		
*Campylobacter* spp.	Camp16SF	CACGTGTCACAATGGCATAT	108	This study
	Camp16SR	GGCTTCATGCTCTCGAGTT		

### *Chlamydiales*-specific real-time PCR

A pan-*Chlamydiales* PCR that may amplify all members of the *Chlamydiales* order including *Chlamydiaceae*, *Waddliaceae* and *Parachlamydiaceae* was performed as described previously by Lienard et al. [27]. The CFX96™ real-time PCR (Biorad) was used to perform the amplification and the detection of DNA with a cycling program of 3 min at 95°C, followed by 50 cycles of 15 s at 95°C, 15 s at 67°C, and 15 s at 72°C. Samples were tested at least in duplicates and were considered negative if no amplification was observed during all 50 cycles. Samples positive for members of the *Chlamydiaceae* family were further tested by corresponding commercial kits.

### Commercial standard kits for the quantification of *C. abortus* and *C. pecorum*

The PrimerDesign genesig Kits for *C. abortus* and *C. pecorum* Genomes are designed for the *in vitro* quantification of these two bacteria genomes. The two kits are targeting the *ompA* gene, allowing specific *in vitro* quantification of these species and detecting no other *Chlamydia*. A highly conserved sequence within the *ompA* gene has previously been shown to be a good target sequence in other clinical real time PCR based studies [28]. The primers and probe sequences in this kit have 100% homology with a broad range of clinically-relevant reference sequences.

### *Listeria monocytogenes* PCR

Real-time PCR was performed on the CFX96™ real-time PCR cycler (Biorad). Each reaction contained 10 µl SsoAdvancedTM SYBR1 Green Supermix (Biorad), 0.6 µM of each primer (Table 1), 2 µl template DNA and nuclease free H_2_O to a final volume of 20 µl. The cycling parameters consisted of 3 min incubation at 95°C followed by 45 cycles of 95°C for 15 s and 60°C for 1 min. A melting curve analysis was performed using the following cycling parameters: 60°C for 30 s, and 5°C temperature changes to the end temperature of 95°C. In all experiments, each PCR run included a negative extraction control (sterile water) and a negative PCR control; containing 2 µl DEPC treated H_2_O instead of DNA extract, to detect possible contamination of DNA. Samples were run in duplicate.

### *Salmonella* spp. PCR

The real-time PCR was performed on the CFX96™ real-time PCR cycler (Biorad). Amplification reactions were carried at a final volume of 25 µl containing 0.2 µM of each primer (Table 1), 12.5 µl of 2× SYBR Permix Ex Taq™ Tli RNaseH Plus (TaKaRa) and 1 µl of genomic DNA. PCR amplification was conducted by incubating the samples at 95°C for 30 s, followed by 40 cycles of 5 s at 95°C and 30 s at 60°C. A melting curve analysis was performed using the following cycling parameters: 60°C for 30 s, and 5°C temperature changes to the end temperature of 95°C. In all experiments, each PCR run included a negative extraction control (sterile water) and a negative PCR control; containing 1 µl DEPC treated H_2_O instead of DNA extract, to detect possible contamination of DNA. Samples were run in duplicate.

### *Coxiella burnetii* PCR

The real-time PCR assay was performed on a CFX96™ real-time PCR cycler (Biorad). The optimal concentration of primers was assessed by testing different concentrations (0.05, 0.15, 0.2, 0.3, and 0.5 µM) and by defining the one that gave the highest recorded fluorescence and the lowest threshold cycle (Ct), defined as the point at which the fluorescence crosses the threshold. Each reaction was run in duplicate in a mastermix containing 10 µl of SsoAdvanced SYBR Green Supermix (Biorad), 0.2 µM of each primer (Table 1), and 1 µl of purified DNA to a final volume of 20 µl. The thermal cycling conditions were assessed by testing different annealing temperatures (between 54°C and 60°C) during different times (5, 10, 20, 30 and 60 s.). The optimal qPCR efficacy was obtained using cycling profile included an initial denaturation step at 95°C for 3 min, then 40 cycles of 10 s at 95°C and 10 s at 60°C. A melting curve analysis was performed using the following cycling parameters: 60°C for 30 s, and 5°C temperature changes to the end temperature of 95°C. In all experiments, each PCR run included a negative extraction control (sterile water) and a negative PCR control (containing 1 µl DEPC treated H_2_O instead of DNA extract, to detect possible contamination of DNA). Samples were run in duplicate.

### *Campylobacter* spp. PCR

The real-time PCR assay was performed on a CFX96™ real-time PCR cycler (Biorad). Each reaction was run in duplicate in a mastermix containing 12.5 µl of 2× SYBR Permix Ex Taq Tli RNaseH Plus (TaKaRa), 0.2 µM of each primer (Table 1), and 1 µl of purified DNA to a final volume of 25 µl using nuclease-free water. The thermal cycling conditions were assessed testing different annealing temperatures (between 54°C and 60°C) during different times (5, 10, 20, 30 and 60 s.). The optimal qPCR efficacy was obtained using cycling profile included an initial denaturation step at 95°C for 30 s, then 40 cycles of 5 s at 95°C and 30 s at 60°C. A melting curve analysis was performed using the following cycling parameters: 60°C for 30 s, and 5°C temperature changes to the end temperature of 95°C. The generated melt peak represented the specific amplified product. In all experiments, each PCR run included a negative extraction control (sterile water) and a negative PCR control (containing 1 µl DEPC treated H_2_O instead of DNA extract, to detect possible contamination of DNA).

### Rose Bengal plate test

All serum samples collected were screened using the Rose Bengal plate test (RBPT), according to the procedures described by Alton et al. [29]. *B. abortus* antigen (obtained from the Institut Pourquier, Montpellier, France) was used to screen sera for the presence of antibodies to *Brucella* spp. In brief, 30 µl of serum was mixed with an equal volume of antigen suspension on a glass plate and agitated. After four minutes of rocking, any visible agglutination was considered as positive. The degree of agglutination was graded on an ordinal scale from 0 (no agglutination) to 3 (coarse clumping), with corresponding RBPT scores of 0, 1, 2 and 3. All doubtful reactions were recorded as negative or zero scores.

### Detection of the amplification product and sequencing

Ten µl of each PCR product were electrophoresed in agarose gel, stained with ethidium bromide and observed under UV illumination. The fragments at correct size were excised and further purified for sequencing. Briefly, DNA was extracted using the QIAquick Gel extraction Kit (Qiagen, Courtaboeuf, France), was then subjected to cycle sequencing using the BigDye Terminator Cycle Sequencing Ready Reaction Kit (Applied Biosystems) and processed by the ABI 3100 Genetic Analyzer. The obtained sequences were compared with the sequences available in GenBank by using the BLAST server from the NCBI website (http://www.ncbi.nlm.nih.gov/BLAST).

### Statistical analysis

The association of abortion with positive results for different agents by real-time PCR and frequencies of positive results between vaginal and milk samples were assessed using the Fisher's exact test. The correlation between real-time PCR and the serological test (RBPT) for *Brucella* cases was assessed using Kruskal-Wallis test (nonparametric test). In all tests, a p value<0.05 was considered to be statistically significant. We decided to use a one-sided p value as all the evidence points to the directionality of this relationship only going one way. All tests were conducted using the Graph-Pad Prism 5 statistical package (Graph-Pad Software, San Diego, CA, USA).

### Ethical considerations

Samples were collected by clinical veterinarians as part of the usual screening scheme on farms and Tunisian ethical guidelines and animal welfare regulations were strictly respected. All herd owners had given an informed consent prior to the study. All samples were collected as part of routine care.

## Results

### Detection of *Brucella* spp

**Figure 1:**
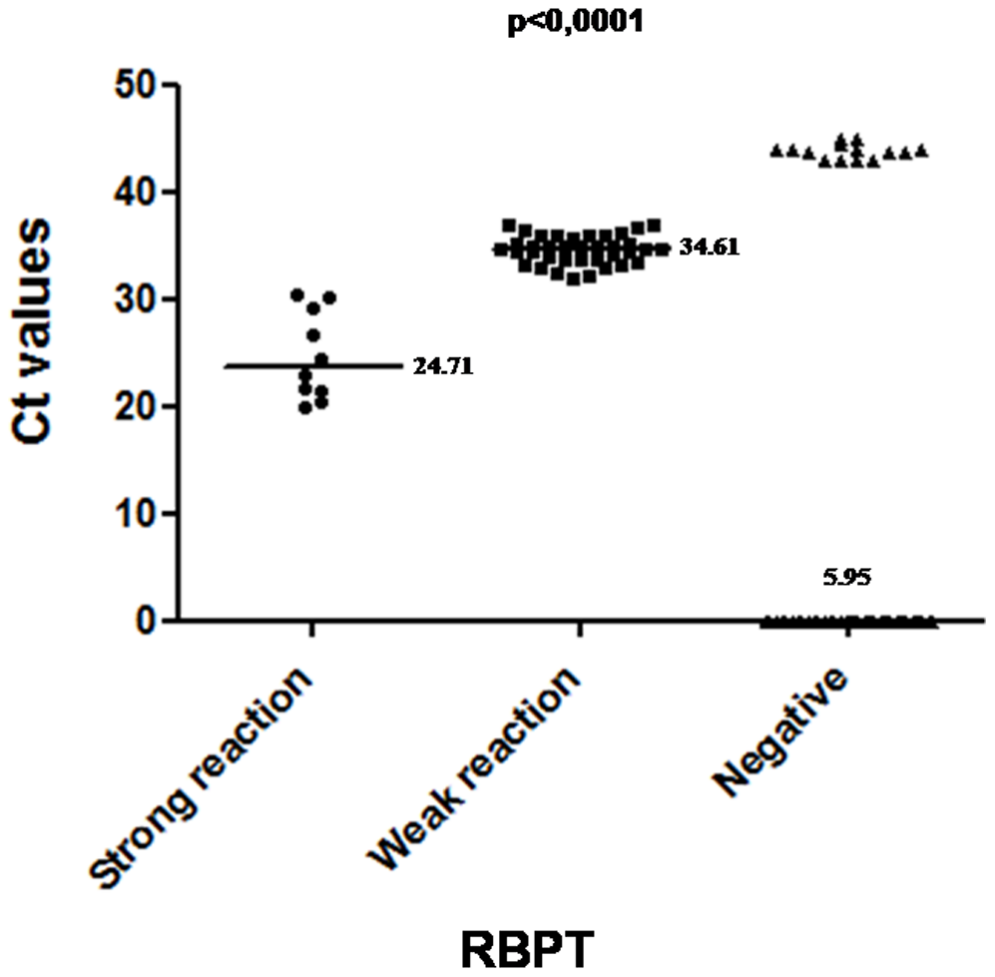
Correlation between real-time PCR and the RBPT results. The mean Ct-value of samples negative for *Brucella* (5.95) was significantly lower than that of samples with a strong reaction results (24.71), which is lower than that of samples with a weak reaction (34.61) (p<0,0001).

The results of this study showed that 47 out of 150 investigated samples (31.3%) were positive for *Brucella* using the RBPT (Table 2). As many as 37 sera showed a weak reaction (1+/2+) whereas 10 sera showed a strong reaction (3+). Only 5 sera were detected as positive among the 64 control sera with a strong reaction (3+) (OR = 5.38, 95% CI [2.02–14.29], p<0.0001) (Table 2; Table 3). The results of the RBPT were confirmed by the real-time PCR performed on vaginal discharges and on milk samples. As many as 46 vaginal swabs out of 150 (30.6%) were positives (Table 3; Fig. S1A). For milk, only 21/150 samples (14%) were found to be positives (OR = 2.71, 95% CI [1.52–4.84], p value = 0.0004) (Table 3; Fig. S2A). All control samples remained negative by real-time PCR. Samples showing Ct values above 43 were considered as negative. Interestingly, the mean Ct-value of samples negative for *Brucella* was significantly lower than that of samples with a strong reactivity, which is lower than that of samples with a weak reactivity (p<0.0001) (Fig. 1). In conventional gel PCR, all positive samples by real-time PCR produced amplicon of 63 bp when resolved on an agarose gel (Figs. S1B, S2A). All of them were identified as *B. abortus* based on species-specific PCR [30]. To confirm that positive samples were due to the presence of *B.* abortus DNA, PCR products were sequenced and analyzed using the BLAST web interface (http://blast.ncbi.nlm.nih.gov/Blast.cgi). The amplicons of these positive samples were found to be 100% identical to the *B. abortus* gene in the database. Details of cases positive for *B. abortus* are given in Table 2.

**Table 2:** Details of cases positive for *Brucella abortus*.

	Number of positive cases	Rose Bengal plate test (RBPT)	Real-time PCR	
			Vaginal swab samples	Milk samples
**Aborted cows**	21	+	+	+
	25	+	+	−
	1	+	−	−
**Control cows**	5	+	−	−

+ positive/ − negative.

**Table 3:** The association of abortion with positive results for different agents and frequencies of positive results between vaginal and milk samples.

Tests	Cases (n = 150)	Controls (n = 64)	Odds Ratio 95% CI	P value
**RBPT**	47 (31.3%)	5 (7.81%)	OR 5.38 (2.02–14.29)	<0.0001
***Brucella*** ** spp. PCR**	46 (30.6%)	0	OR 57.4 (3.47–948.3)	<0.0001
***Chlamydiales*** ** PCR**	27 (18%)	0	OR 28.7 (1.72–478.9)	<0.0001
***L. monocytogenes*** ** PCR**	7 (4.6%)	0	OR 6.74 (0.37–119.9)	0.0796
***Salmonella*** ** spp. PCR**	5 (3.3%)	0	OR 4.87 (0.26–89.5)	0.1658

Tests	Vaginal swab samples (n = 150)	Milk samples (n = 150)	Odds Ratio 95% CI	P value
***Brucella*** ** spp. PCR**	46 (30.6%)	21 (14%)	OR 2.71 (1.52–4.84)	0.0004
***Chlamydiales*** ** PCR**	27 (18%)	0	OR 67 (4.04–1111)	<0.0001
***L. monocytogenes*** ** PCR**	4 (2.6%)	3 (2%)	OR 1.34 (0.29–6.1)	0.5
***Salmonella*** ** spp. PCR**	0	5 (3.3%)	OR 0.08 (0–1.6)	0.0302

The association of abortion with positive results for different agents by real-time PCR and frequencies of positive results between vaginal and milk samples were assessed using the Fisher's exact test. A p value<0.05 was taken as a level of significance. Cases: cows with clinical signs, controls: cows with normal pregnancies and normal parturitions, OR: odds ratio, CI: confidence interval.

### Detection of *Chlamydiaceae, W. chondrophila, P. acanthamoebae*

*Chlamydial* DNA was detected in 27 (18%) vaginal swab samples, all obtained from the 150 cows that suffered from an abortion (OR = 28.7, 95% CI [1.72–478.9], p<0.0001). Briefly, these cows exhibited symptoms of reproductive disorders like abortion and other symptoms like fever, appetite loss, weakness and diarrhea. No milk sample was positive (OR = 67, 95% CI [4.04–1111], p<0.0001). In conventional gel PCR, the 27 vaginal swab samples positive by real-time PCR produced as expected amplicons of 207 bp when resolved on agarose gel 1.5% (Fig. S3A, S3B). PCR products were sequenced and analyzed using the BLAST web interface (http://blast.ncbi.nlm.nih.gov/Blast.cgi). Among 27 sequences obtained, 12 (8%) belonged to the *Waddliaceae* family, 8 (5.3%) belonged to the *Parachlamydiaceae* family and 7 (4.6%) belonged to the *Chlamydiaceae* family (Fig. S3A, S3B). All sequences corresponding to a *Waddliaceae* species demonstrated ≥99% similarity with *W. chondrophila*. All of them had already been identified by the SYBR Green real-time PCR we used in our previous work [12] to detect the presence of *W. chondrophila*. Since both PCR are targeting two different DNA regions, these results may clearly not result from PCR contamination with amplicons. All the sequences belonging to *Parachlamydiaceae* family demonstrated ≥99% of similarity with *P. acanthamoebae*. Four among the 7 sequences belonging to the *Chlamydiacaeae* family demonstrated ≥99% sequence similarity with *C. abortus*, whereas the remaining 3 sequences exhibited ≥99% sequence similarity with *C. pecorum*. These results were confirmed by two commercial kits (PrimerDesign genesig) specific for the quantification of *C. abortus* and *C. pecorum*. All control samples remained negative by real-time PCR. Details of cases positive for *P. acanthamoebae, C. abortus, C. pecorum* and *W. chondrophila* are given in Table 4.

**Table 4:** Sequencing results of vaginal swab samples positive with the pan-*Chlamydiales* PCR.

Animal no.	Age (years)	Other information	Other etiology	Ct values (mean values)	% 16S rRNA gene homology with most similar GenBank sequence (corresponding family)
**1**	2	Abortion, fever, loss of appetite, diarrhea	*Brucella abortus*	28.34	99% *Parachlamydia acanthamoebae*
**2**	2	Abortion, fever, diarrhea		34.91	99% *Parachlamydia acanthamoebae*
**3**	3	Abortion, fever, weakness, diarrhea		34.91	*99% Parachlamydia acanthamoebae*
**4**	2	Abortion, fever, diarrhea	*Brucella abortus*	35.72	99% *Parachlamydia acanthamoebae*
**5**	2	Abortion		31.8	99% *Parachlamydia acanthamoebae*
**6**	2	Abortion	*Brucella abortus*	34.52	99% *Parachlamydia acanthamoebae*
**7**	2	Abortion, fever, diarrhea		35.22	99% *Parachlamydia acanthamoebae*
**8**	2	Abortion, fever, diarrhea		35.64	99% *Parachlamydia acanthamoebae*
**9**	4	Abortion, fatigue	*Brucella abortus*	19.97	99% *Chlamydia abortus*
**10**	3	Abortion, respiratory disease	*Brucella abortus*	22.91	99% Chlamydia abortus
**11**	4	Abortion		25.4	99% Chlamydia abotus
**12**	2	Abortion	*Brucella abortus*	25.64	99% Chlamydia abortus
**13**	2	Abortion, diarrhea, fatigue		27.79	99% *Chlamydia pecorum*
**14**	3	Abortion		31.66	99% *Chlamydia pecorum*
**15**	2	Abortion, loss appetite		34.52	99% *Chlamydia pecorum*
**16** *	2	Abortion	*Listeria monocytogenes*	32.88	99% *Waddlia chandrophila*
**17** *	2	Abortion, fatigue		35.49	99% *Waddlia chandrophila*
**18** *	2	Abortion, fever, loss appetite, weakness, diarrhea		29.72	99% *Waddlia chandrophila*
**19** *	3	Abortion		34.26	99% *Waddlia chandrophila*
**20** *	3	Abortion, respiratory diseases		33.24	99% *Waddlia chandrophila*
**21** *	2	Abortion, diarrhea		31.33	99% *Waddlia chandrophila*
**22** *	3	Abortion		34.1	99% *Waddlia chandrophila*
**23** *	3	Abortion	*Listeria monocytogenes*	36.1	99% *Waddlia chandrophila*
**24** *	4	Abortion, fever, loss appetite		31.97	99% *Waddlia chandrophila*
**25** *	2	Abortion		34.62	99% *Waddlia chandrophila*
**26** *	2	Abortion		33.83	99% *Waddlia chandrophila*
**27** *	4	Abortion, fever, diarrhea		32.84	99% *Waddlia chandrophila*

*These results were previously published by Barkallah et al. (2013).

### Detection of *L. monocytogenes*

*L. monocytogenes* DNA was detected in 7/150 (4.66%) vaginal swab samples all obtained from the 150 cows that suffered of an abortion (OR = 6.74, 95% CI [0.37–119.9], p value = 0.08) (Table 3). Only 3/150 (2%) milk samples were found to be positive (Table 3). These cases originated from different herds in different regions of Sfax. All control samples remained negative by real-time PCR. The real-time PCR melt curve data identified one peak with a melting temperature of 87°C for all samples (Fig. S4A, S4B). In conventional gel PCR, all samples positive by real-time PCR produced amplicon of 63 bp when resolved on an agarose gel 3% (Fig. S4A, S4B). To confirm that positive samples were due to the presence of *L. monocytogenes* DNA, PCR products were sequenced and analyzed using the BLAST web interface (http://blast.ncbi.nlm.nih.gov/Blast.cgi). The amplicons of these positive samples were 100% identical to the *L. monocytogenes hlyQ* gene in the databases. All control samples remained negative by real-time PCR.

### Detection of *Salmonella* spp

*Salmonella* DNA was detected in 5 (3.33%) milk samples all obtained from the 150 cows that suffered of an abortion (OR = 4.87, 95% CI [0.26–89.5], p value = 0.1658) (Table 3). No salmonella DNA was detected in vaginal swab samples (OR = 0.08, 95% CI [0–1.6], p value = 0.0302) (Table 3). These cases were collected from the same herd located in the southern region of Sfax. The real-time PCR melt curve data identified one peak with a melting temperature of 76°C for all samples (Fig. S5A). In conventional gel PCR, all positive samples by real-time PCR produced amplicon of 243 bp when resolved on an agarose gel 1.5% (Fig. S5B). To confirm that positive samples were due to the presence of *Salmonella* spp. DNA, PCR products were sequenced and analyzed using the BLAST web interface (http://blast.ncbi.nlm.nih.gov/Blast.cgi). The amplicons of these positive samples were 100% identical to *Salmonella enterica* serovar Typhimurium in the databases.

### Detection of *C. burnetii* and *Campylobacter* spp

*C. burnetii* was absent in all bovine samples taken from the 150 cows that have aborted and from the 64 control cows tested by the real-time PCR. Similarly, using the *Campylobacter* spp. real-time PCR, none of the bovine samples taken from the 150 cows that have aborted and from the 64 control cows, were positive.

### Co-infections

From a total of 150 abortion cases collected, infectious agents were detected in 73 (48.66%) cases, 13 (8.66%) of which represented co-infections with two infectious agents. These observations may suggest that co-infections commonly exist in cattle herds. Table 5 shows the number of positive samples for each pair of pathogens.

**Table 5:** The number of samples positive for each pair of pathogens.

	*B. abortus*	*C. abortus*	*C. pecorum*	*W. chondrophila*	*P. acanthamoebae*	*L. monocytogenes*	*Salmonella* Typhimurium	*Campylobacter* spp.	*C. burnetii*
***B. abortus***	47	3	0	0	3	0	5	0	0
***C. abortus***	3	4	0	0	0	0	0	0	0
***C. pecorum***	0	0	3	0	0	0	0	0	0
***W. chondrophila***	0	0	0	12	0	0	0	0	0
***P. acanthamebea***	3	0	0	0	8	0	0	0	0
***L. monocytogenes***	0	0	0	2	0	7	0	0	0
***Salmonella*** ** Typhimurium**	5	0	0	0	0	0	5	0	0
***Campylobacter*** ** spp.**	0	0	0	0	0	0	0	0	0
***C. burnetii***	0	0	0	0	0	0	0	0	0

## Discussion

In many developing countries, studies conducted to estimate the true prevalence of abortigenic agents were only occasional and limited to certain farms or regions. Consequently, the control programs are not very effective, even for established agents of abortion in cattle. Moreover, the etiology remains unknown in many cases of bovine abortion. In this study, we thus focused on new or not well-defined emerging agents in Tunisian cattle. Vaginal swab samples were used in many previous studies for the detection and isolation of many abortive bacteria [28], [31]–[35], because this type of sampling (i) is simple, fast and it is not necessary to have access to the placenta and (ii) limits the external contamination, which is often the case in the collection of the cotyledons if the placenta hit the ground [36]. The choice of milk specimens is based on the fact that milk is the most frequent shedding route in chronically infected cows [37]–[39] and the transmission of infection to humans is mainly due to the consumption of raw milk and dairy products [39]–[43].

This work studies the etiological agents of some infectious causes of bovine abortion during mid- to late-gestation in Tunisian dairy herds. The high percentage of positive cases for *B. abortus* detected in the present study both through RBPT (31.3%) and PCR (30.6%) was unexpected. Quantitatively, the current results are relatively similar to other studies based on the detection of *Brucella* in African veterinary samples by serology tests [44]–[46] and by the IS711 real-time PCR [47]. There is a strong correlation between the DNA copies (Ct values) obtained using the real-time PCR on vaginal swab samples and the reactions intensity obtained by the RBPT (p<0.0001) as previously reported by Abdalla and Hamid [47] on Sudanese bovine samples. Using RBPT, five controls were diagnosed as positive versus none by real-time PCR. This difference between the two tests was not significant (p value = 0.058). Incorrect timing or different site of sampling may explain observed false negative results. RBPT may also give false positive results because (i) the antibody response to vaccination cannot be differentiated from the one observed after field infection and (ii) cross-reactivity seen with other bacteria are a well-known problem in serological diagnosis of brucellosis. The real-time PCR set up in this study detected more cases of bovine brucellosis in vaginal swab samples (30.6%) than in milk (14%). Our results suggest that *Brucella* may be more readily detected in vaginal samples than in milk (30.6% vs 14%, p value = 0.0004) (Table 3), a statistically significant finding. Reports on the ability to detect *Brucella* spp. in milk samples from infected animals by PCR can be somewhat conflicting when compared to standard serological [48] and bacteriological methods. Thus, the results of our study confirm that milk is not the best sample to detect *Brucella* DNA. No correlation was found between *B. abortus* positivity and age, herd size and breeding system. Considering abortion as the only clinical sign, there was an association between the occurrence of abortion and positive results by the RBPT (31.3% vs 7.81%, p<0.0001) and real-time PCR (30.6% vs 0%, P<0.0001) (Table 3), a statistically significant and clinically important finding. The percentage of *Brucella* contamination showed that brucellosis is endemic in cows from different regions of Sfax, despite the extensive vaccination programs implemented as one of the main control measures.

Chlamydiosis seem also to play an important role in bovine abortion in Tunisia, since twenty-seven samples out of 150 (18%) were found to be positive by real-time PCR. The combination of real-time PCR and two commercial diagnostic kits identified 7 positive cases for *C. abortus* (n = 4) and *C. pecorum* (n = 3). Using the specific real-time PCR, three samples were shown to be infected by both *C. abortus* and *B. abortus*. The association of these two known abortigenic agents was not described yet. In these cases of mixed infection, it remains unclear if both or only one is the etiological agent of bovine abortion. The 3 cases positive for *C. pecorum* were negatives for all other agents investigated in this study. *C. pecorum* is known as the causing agent of conjunctivitis, arthritis, pulmonary inflammation and fertility disorders in Tunisian ruminants and could also be detected in ovine placenta possibly leading to abortion [49]. On the other hand, *C. pecorum* occurs in asymptomatic gastrointestinal infections of ruminants. Thus, a transmission of infection from sheep to cattle cannot be excluded especially in areas where there is a contact between the two animal species. *Chlamydia*-related organisms seem to play an important role in bovine abortion since 20 samples were positive (13.3%). Similar results were given by Swiss studies based on the detection of *Chlamydia*-related organisms in veterinary samples using another PCR [50]. The involvement of *W. chondrophila* in abortion in Tunisia was previously confirmed in a previous work [12]. In the present study, 8 (5.33%) bovine vaginal samples were also positive for *P. acanthamoebae* in the subset of 150 samples. The results for all 8 positive samples were confirmed by the *P. acanthamoebae* PCR previously described by Casson et al. [51]. This is the first description of this *Chlamydia*-related organism in bovine abortion in African cattle. All positive samples were collected from cows belonging to a same herd including 1500 dairy cows. The percentage of abortion in this herd was 12% during 2011 knowing that all cows in this herd were vaccinated against *Brucella*, Bovine Viral Diarrhoea (BVD) and Infectious Bovine Rhinotrachetis (IBR) viruses. These 8 samples were however negatives for *C. abortus*, *C. pecorum*, *B. melitensis*, *C. burnetii*, *L. monocytogenes*, *Salmonella* spp., *Campylobacter* spp., BVD and IBR Viruses. The co-infection of *P. acanthamoebae and B. abortus* (three cases) has not been described yet. Again it remains unclear if both or only one agent caused bovine abortion. For the five remaining cases, no other agents have been detected and no other conditions explaining abortion were documented. Thus, our data suggest that *P. acanthamoebae* is a possible factor in some cases of abortion. This is consistent with a Swiss work that identified *P. acanthamoebae* in placenta of aborted bovines by PCR, immunohistochemistry and electron microscopy [13]. Based on the present work, *Chlamydia* and other *Chlamydia*-related bacteria do not appear to be excreted in the milk of cows. This is in concordance with the reports of other authors stating that shedding of the *C. abortus* in milk is possible but uncommon [52]. It is likely that the excretion of *Chlamydia* organisms was more strongly manifested with vaginal discharge than with milk (18% vs 0%, p<0.0001), a statistically significant and clinically important finding.

*L. monocytogenes* is another agent that may be involved in bovine abortion. This is the first time that bovine abortion samples from Tunisia were investigated for *L. monocytogenes*. Besides the 2 cases of mixed infection with *L. moncytogenes* and *W. chondrophila* already described above [12], 5 new cases positive for *L. monocytogenes* were found, in which no other agents have been detected. There was no association between the occurrence of abortion and positive results for this bacterium by real-time PCR (4.6% vs 0%, p value = 0.08). In addition, our results suggest that *Listeria* may be readily detectable in vaginal samples and also in milk (2.6% vs 2%, p value = 0.5). Infection with *L. monocytogenes* is transmissible to humans through milk and milk products [53], in which case it can cause systemic and often fatal diseases. There are other agents such as *Salmonella* that may be involved in cattle abortion and can be transmitted to humans through the consumption of milk. In this study, *S. enterica* Typhimurium was detected only in 5 milk samples taken from 5 cows that suffered an abortion. In these 5 cases, *B. abortus* DNA was also detected by a specific real-time PCR in vaginal and milk samples. When mixed infection occurs, it remains unclear if both or only one agent caused bovine abortion. If we consider abortion as the only clinical sign, there is no association between the occurrence of abortion and positive cases for *Salmonella* by real-time PCR (3.3% vs 0%, p value = 0.1658). In many previous studies, it was revealed that abortion was a quite unusual clinical finding of *S. enterica* Typhimurium infection in cattle because it hasn't a higher potential for systemic dissemination in body [54]. *S. enterica* Typhimurium is often associated with enteritis that usually affects young calves, resulting in marked acute diarrhea [55]. All these data suggest that *B. abortus* may be the most probable agent of abortion in these 5 cows belonging to the same herd located in the south of Sfax. Unlike to other agents cited above, *Campylobacter* spp. and *C. burnetii* were not detected in the limited number of cases studied. This is in contrast with previous studies in ruminants and human beings that supported a role of *C. burnetii* in different mammals. In fact, the seroprevalence of Q fever in sheep flocks in different Tunisian regions was 40% and *C. burnetii* was the abortigenic agent in 17% of sheep and goats [56]. *C. burnetii* does not seem to have a crucial role in bovine abortion in contrast to the small ruminants. Previous studies already showed negligible percentages for the involvement of *Campylobacter* spp. in bovine abortion [34], [57]. More samples should be collected to survey the infections caused by these two bacteria in dairy herds.

This study highlighted the real prevalence of brucellosis and the presence of diseases that are not subject to routine control diagnosis, such as chlamydiosis, salmonellosis and listeriosis. It also demonstrated the importance of *Chlamydia and Chlamydia*-related organisms as major causes of abortion. Strong evidence suggests that *P. acanthamoebae* should be considered as a new abortigenic agent in Africa. As well, this study underlined the notion of co-infection, which emphasizes the great interest of a differential diagnosis of different abortion causes.

## Supporting Information

Figure S1 ***Brucella*** **spp. real-time PCR, direct amplification from bovine vaginal samples.**
(TIF)

Figure S2 ***Brucella*** **spp. real-time PCR, direct amplification from bovine milk samples.**
(TIF)

Figure S3 **Pan-** ***Chlamydiales*** **real-time PCR, direct amplification from bovine vaginal samples.**
(TIF)

Figure S4 ***Listeria monocytogenes*** **real-time PCR melt curve data and 3% agarose gel images for determining primer specificity and product size.**
(TIF)

Figure S5 ***Salmonella*** **spp. real-time PCR melt curve data and 1.5% agarose gel image for determining primer specificity and product size.**
(TIF)
