# Supplementary figures and images for: Survey of Infectious Etiologies of Bovine Abortion during Mid- to Late Gestation in Dairy Herds
Source: PLoS One. 2014 Mar 24;9(3):e91549. doi: 10.1371/journal.pone.0091549 (PMC3963856; doi:10.1371/journal.pone.0091549)

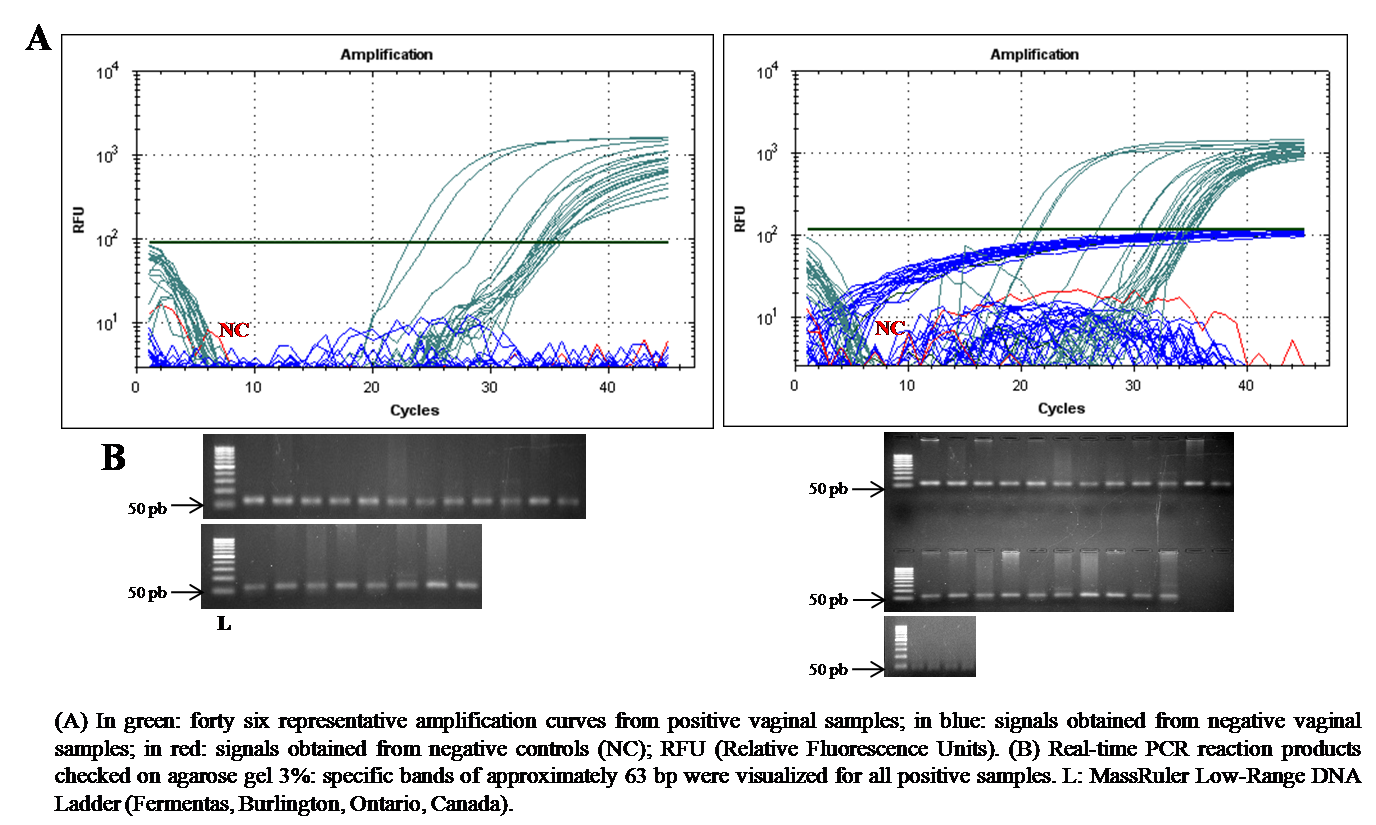

Supplement: Figure S1 — Brucella spp. real-time PCR, direct amplification from bovine vaginal samples. (TIF) [file pone.0091549.s001.tif]

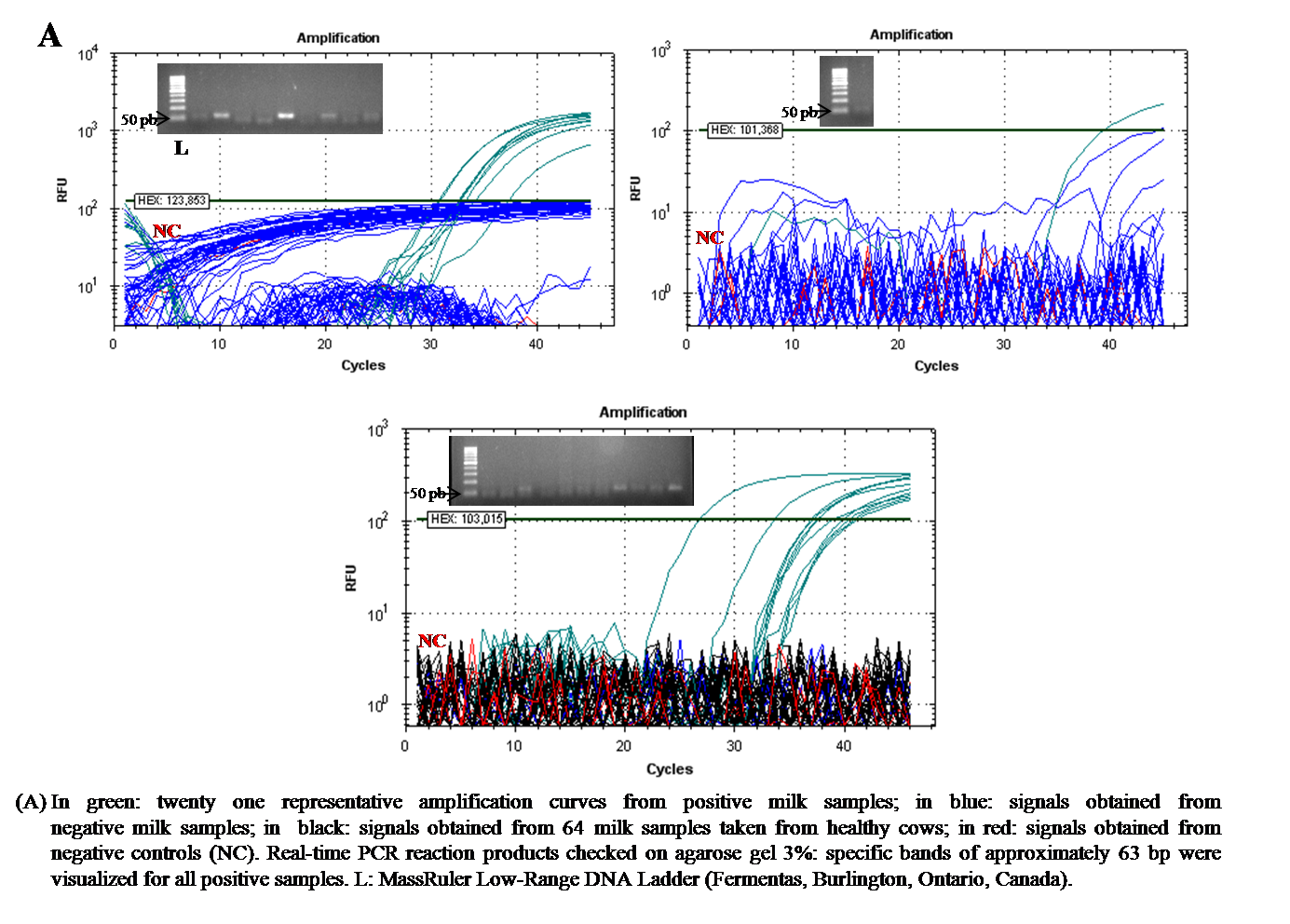

Supplement: Figure S2 — Brucella spp. real-time PCR, direct amplification from bovine milk samples. (TIF) [file pone.0091549.s002.tif]

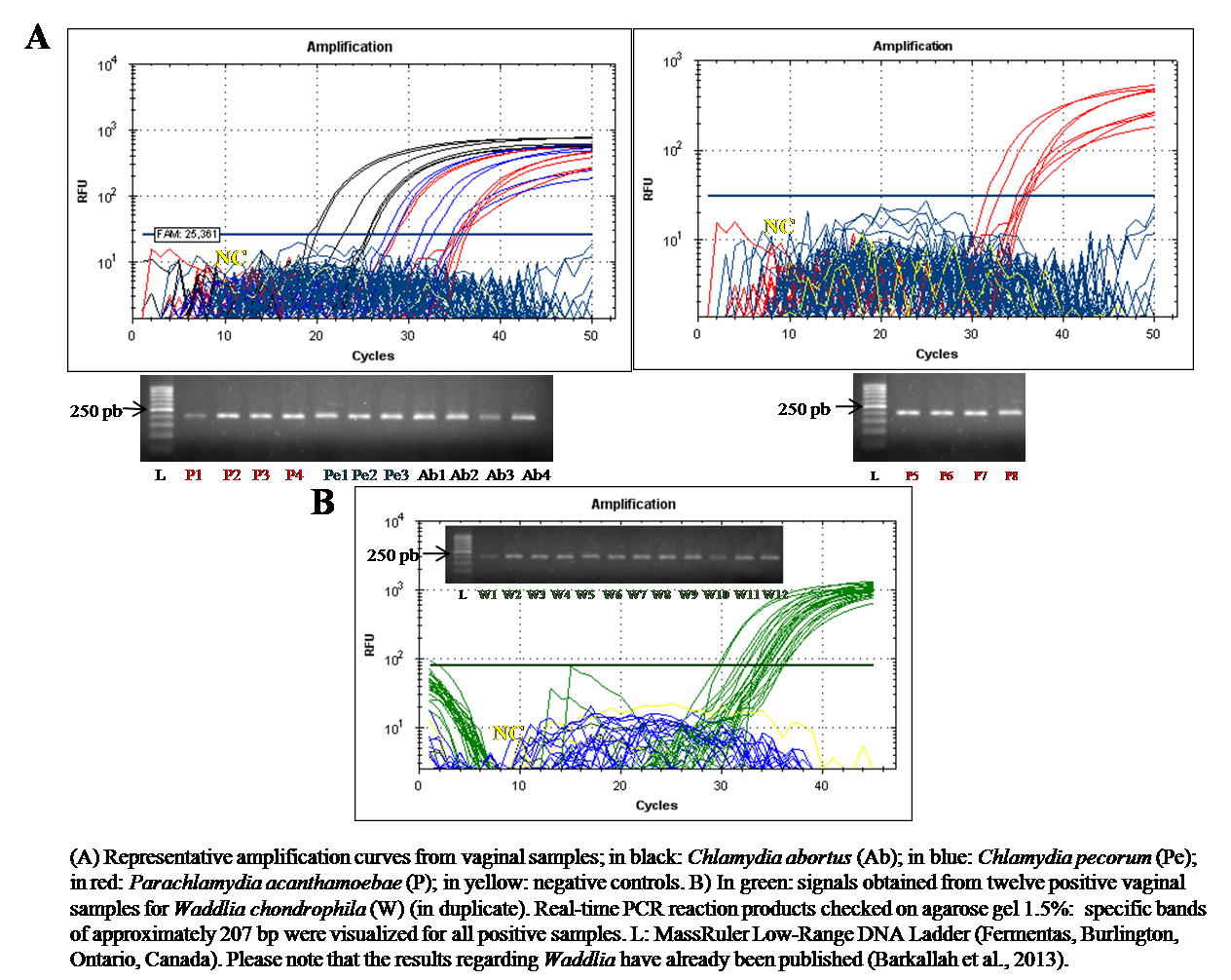

Supplement: Figure S3 — Pan- Chlamydiales real-time PCR, direct amplification from bovine vaginal samples. (TIF) [file pone.0091549.s003.tif]

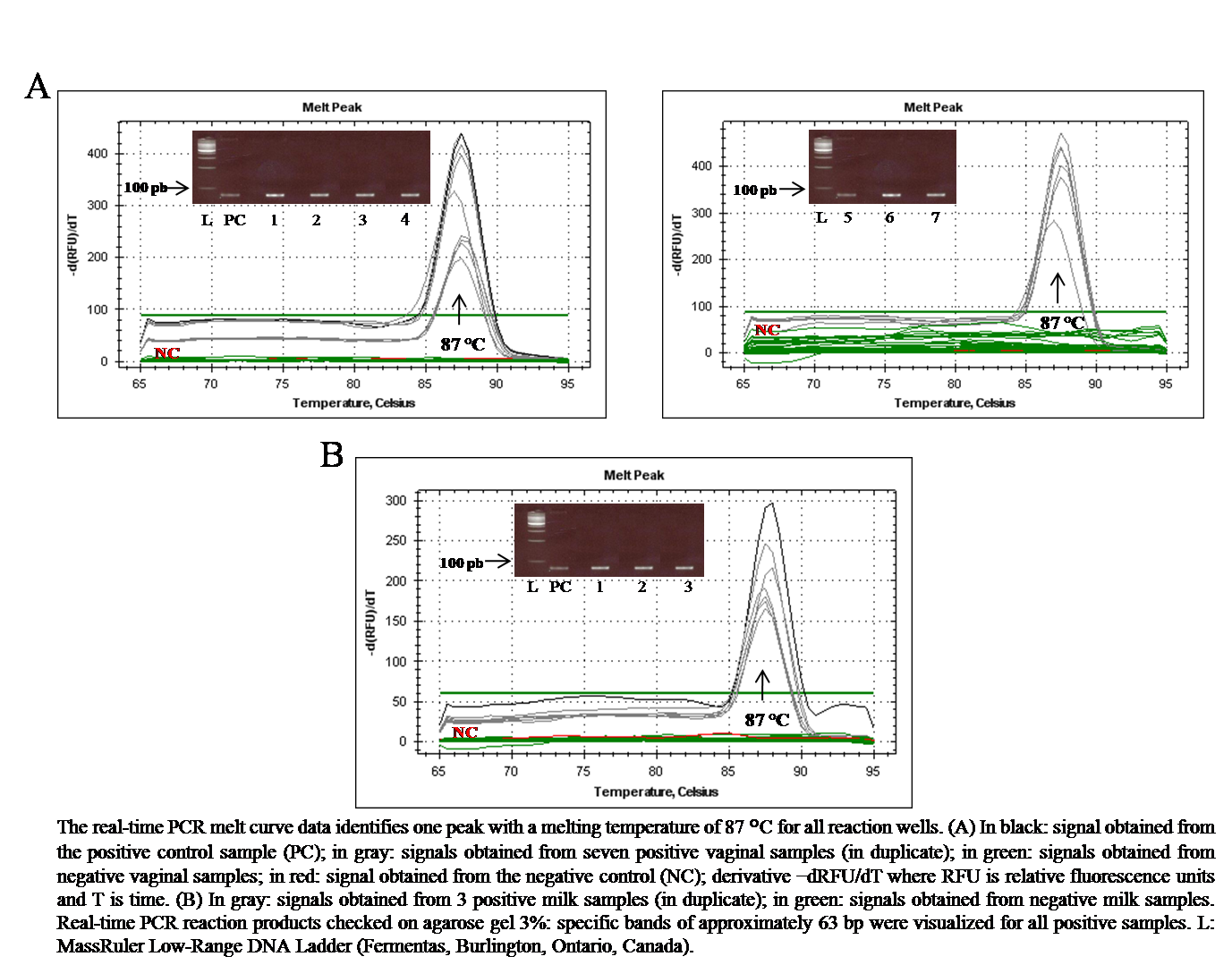

Supplement: Figure S4 — Listeria monocytogenes real-time PCR melt curve data and 3% agarose gel images for determining primer specificity and product size. (TIF) [file pone.0091549.s004.tif]

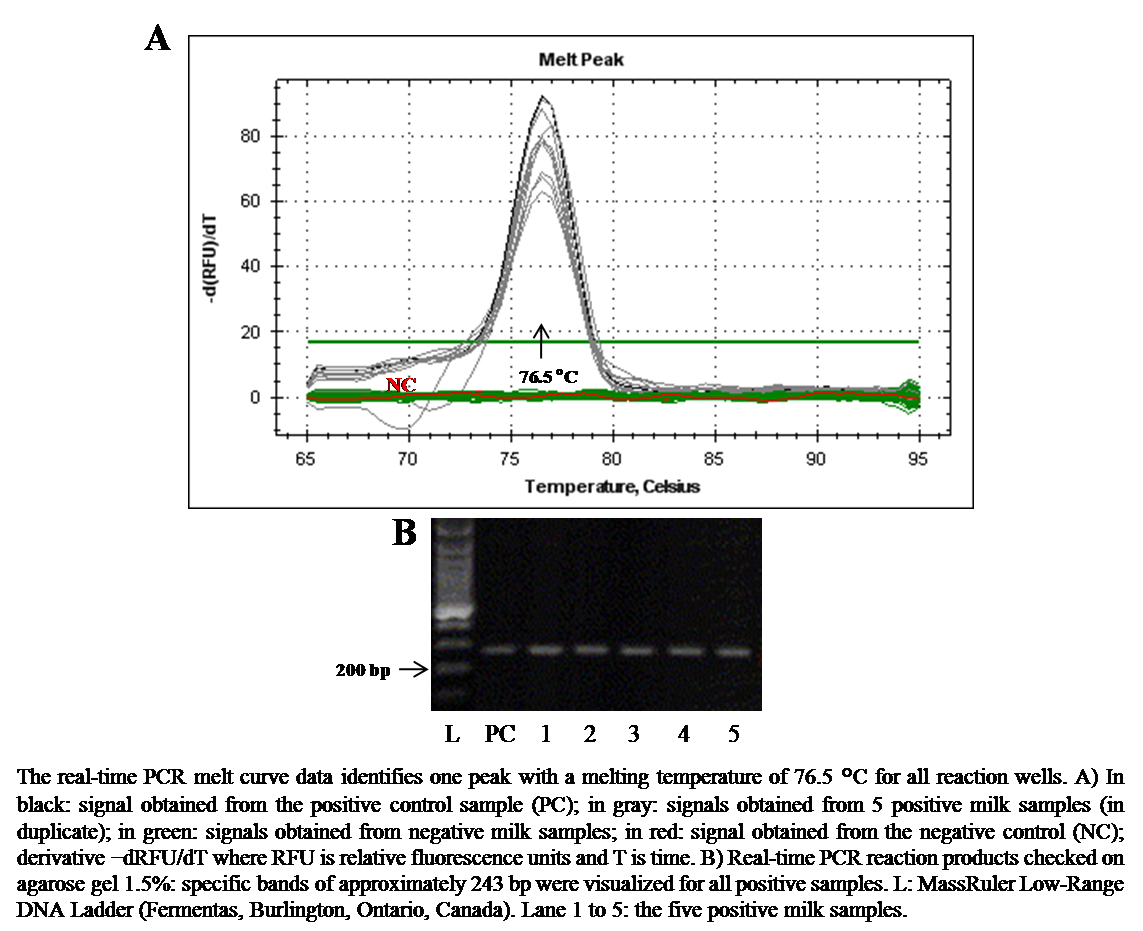

Supplement: Figure S5 — Salmonella spp. real-time PCR melt curve data and 1.5% agarose gel image for determining primer specificity and product size. (TIF) [file pone.0091549.s005.tif]
